# Magnesium for Pain Treatment in 2021? State of the Art

**DOI:** 10.3390/nu13051397

**Published:** 2021-04-21

**Authors:** Véronique Morel, Marie-Eva Pickering, Jonathan Goubayon, Marguérite Djobo, Nicolas Macian, Gisèle Pickering

**Affiliations:** 1 Clinical Investigation Center, Inserm CIC 1405, Bat 3C, University Hospital Clermont-Ferrand, F-63000 Clermont-Ferrand, France; v_morel@chu-clermontferrand.fr (V.M.); jgoubayon@chu-clermontferrand.fr (J.G.); mdjobo@chu-clermontferrand.fr (M.D.); nmacian@chu-clermontferrand.fr (N.M.); 2 Rheumatology Department, University Hospital Clermont-Ferrand, F-63000 Clermont-Ferrand, France; mepickering@chu-clermontferrand.fr

**Keywords:** magnesium, pain, comorbidity, randomised clinical trial

## Abstract

Background: Magnesium (Mg) is commonly used in clinical practice for acute and chronic pain and has been reported to reduce pain intensity and analgesics consumption in a number of studies. Results are, however, contested. Objectives: This review aims to investigate randomised clinical trials (RCTs) on the effectiveness of Mg treatment on pain and analgesics consumption in situations including post-operative pain, migraine, renal pain, chronic pain, neuropathic pain and fibromyalgia. Results: The literature search identified 81 RCTs (*n* = 5447 patients) on Mg treatment in pain (50 RCTs in post-operative pain, 18 RCTs in migraine, 5 RCTs in renal pain, 6 RCTs in chronic/neuropathic pain, 2 RCTs in fibromyalgia). Conclusion: The level of evidence for the efficacy of Mg in reducing pain and analgesics consumption is globally modest and studies are not very numerous in chronic pain. A number of gaps have been identified in the literature that need to be addressed especially in methodology, rheumatic disease, and cancer. Additional clinical trials are needed to achieve a sufficient level of evidence and to better optimize the use of Mg for pain and pain comorbidities in order to improve the quality of life of patients who are in pain.

## 1. Introduction

Pain, acute or chronic, affects a large number of individuals worldwide. The physiology of pain is complex, with activation of nociceptors, transduction of nervous signals, ascending pathways transmission and modulation of pain in the descending inhibitory pathways [1,2,3]. Pain involves not only sensori-discriminative, but also cognitive, emotional, behavioural and social dimensions. Chronic pain [3] affects a large number of persons, with a prevalence of 33.2% in the general population [4] and is accompanied by a number of comorbidities like stress that may be amplified in a vicious circle [4,5]. Among common comorbidities, migraine [6], anxiety and depression [7], sleep disorders [4] and impaired quality of life [8] are frequently described.

Magnesium (Mg) is often used in the community by healthy persons and patients with pain [9] as a supplementary drug to improve their well-being [10] and reduce stress [11]. This use is facilitated by its over-the-counter availability and many pharmaceutical presentations are available [12]. It is also commonly used in hospital for pain management, alone, or in combination with analgesics like morphine [13] or ketamine, an antihyperalgesic agent and N-methyl-D aspartate receptor (NMDAR) antagonist [14,15], that may also improve stress and depressive symptoms [16,17].

The frequent use of Mg in painful acute situations like post surgery, or in chronic pain, relies on the fact that Mg is the physiological blocker of the NMDAR. At a neuronal level it plays a major role in controlling the excitability of NMDAR [18] as it is a constitutive antagonist of this receptor [19]. Central sensitisation of pain and long-term potentiation (LTP) are related to hyperexcitability at the level of the NMDAR, a ubiquitous receptor that plays a pivotal role in the chronicisation of pain but also on learning and cognitive processes. NMDAR is widely localised in the central nervous system, including the hippocampus, anterior cingulate cortex, insular cortex and dorsal horn of the spinal cord [20]. NMDAR opening is triggered by the influx of pre-synaptic glutamate, but also by post-synaptic depolarization (normally caused by the activation of glutamate-sensitive AMPA (α-amino-3-hydroxy-5-methyl-4-isoxazolepropionic acid) receptors). Glutamate binding forces a conformational change by induced adjustment of the NMDAR that opens the pore and releases Mg if there is a depolarization that repels it [21]. NMDAR allows the entry of calcium into the cell and induces modulation of the intensity of the synaptic transmission force [22]. Furthermore, several intracellular cascades are involved in LTP particularly via the activation of CAM kinase II by calcium [23]. These molecular cascades are described in pain but also suggested in the occurrence of sleep disorders [24], anxiety [25] and fatigue [26].

Considering that the use of Mg for pain treatment has become a fairly common practice in various acute and chronic pain situations, the objective of this paper aims to review publications and randomised clinical trials (RCTs) of Mg in pain to identify the impact of Mg on pain relief and analgesics reduction in painful situations.

## 2. Materials and Methods

The Medline^®^, Pubmed^®^, Google Scholar and Cochrane databases were searched until March 2021 to identify reviews and RCTs using the keywords “magnesium AND pain”, “magnesium”, “analgesics AND magnesium”. Several pieces of information were collected including study design, number of subjects, control group, pain aetiology, Mg administration protocol, primary endpoint and results. The parameters necessary to retain these randomised studies were the evaluation of pain following administration of Mg and/or the analgesics consumption; there was no age limit nor a minimum number for the population, no specific requirement regarding the years of publication and studies had to be available in English. RCTs that did not address these parameters were discarded. In addition, our search included publications on the bioavailability of the different Mg salts in order to identify specificities among pharmaceutical preparations.

## 3. Results

**Figure 1:**
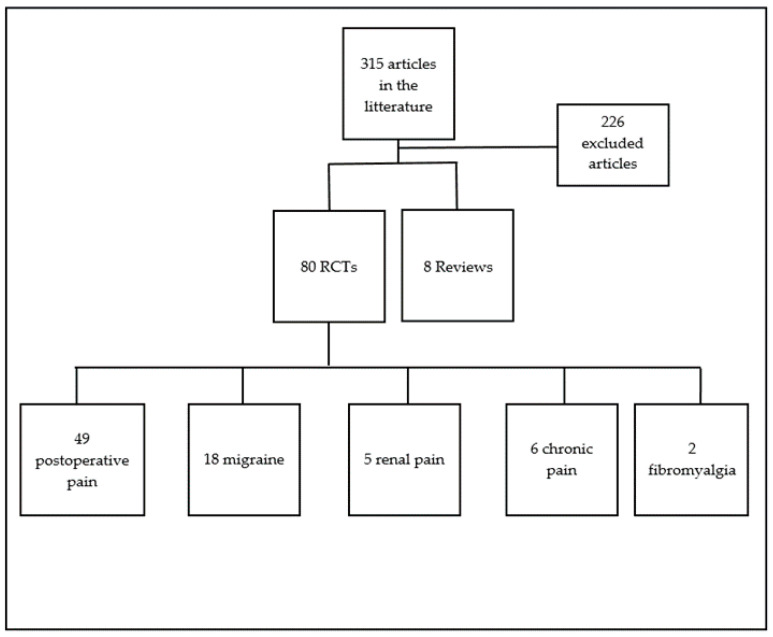
Flow chart of articles on magnesium, pain and analgesics consumption.

A total of 315 articles were identified; 226 articles were discarded (not conforming to the inclusion criteria). Eighty RCTs and 8 systematic reviews [10,13,27,28,29,30,31,32] were appropriate for this review (adequacy of the abstract with the review: exploration of the efficacy of Mg in pain and consumption of analgesics) (Figure 1). Pain reduction was assessed by visual analogue scale (VAS) (0 no pain—10 (or 100) worst possible), and evaluated at different times, or with questionnaires specific to the pathology as described further. The effect of Mg on pain was studied in 75/80 RCTs (*n* = 4981) and on analgesics consumption in 51/80 RCTs (*n* = 3656). Analgesics consumption was described as a qualitative increase or diminution of analgesics. In addition, the review retrieved several articles on Mg salts bioavailability, assessed by the percentage of absorption of the salts.

### 3.1. Magnesium and Pain Diminution

In post-operative pain, 49 RCTs studied the effectiveness of Mg in reducing pain (VAS) and/or on analgesics consumption (40 RCTs explored both parameters, 4 explored pain evolution only and 5 analgesics use only) [33,34,35,36,37,38,39,40,41,42,43,44,45,46,47,48,49,50,51,52,53,54,55,56,57,58,59,60,61,62,63,64,65,66,67,68,69,70,71,72,73,74,75,76,77,78,79,80,81]; 44/49 RCTs explored the efficacy of Mg on the evolution of post-operative pain [33,34,35,36,37,38,41,42,43,44,45,46,47,49,50,51,52,53,54,55,56,57,58,59,61,62,63,64,66,67,68,69,70,71,72,73,74,75,76,77,78,79,80,81] (*n* = 2988). Twenty-nine studies observed a significant decrease of VAS post-operative pain following intravenous administration of Mg sulphate (ranging from –2/10 [36] to −4/10 [58] at 12 h) compared to placebo or conventional treatment group [33,34,35,36,37,41,43,45,46,47,49,51,52,53,54,55,58,59,63,64,67,68,69,71,74,76,78,79,80]. Among the RCTs that showed a significant pain reduction, 6 different procedures were applied, ranging from a simple infusion without bolus to 50 mg/kg with bolus on different types of surgery.

Sixteen RCTs used Mg sulphate vs. placebo with no bolus and different infusion doses. With a 8 mg/kg/h infusion (until the end of the surgical procedure) [35], a significant difference, 12 h after surgery in 60 subjects was observed. With an infusion of 15 mg/kg/h in 40 subjects (for 24 h) [34], a significant pain reduction 12 h after surgery was obtained. A 50 mg/kg infusion during surgery in, respectively, 40 [45] and 83 [47] subjects (for 24 h [45,47]) showed a decrease in pain at 12 h [47] and 24 h [45]. Likewise, during surgery, a 65 mg/kg infusion in 38 subjects (for 12 h) [43] showed a diminution of pain at 2 h and at 4 h but not at 8 h or 12 h.

Thirty-three RCTs used Mg sulphate vs. placebo with a bolus and an infusion. In 36 subjects with a bolus of 20 mg/kg followed by an infusion of 2 mg/kg/h over the total duration of the surgical procedure, a pain decrease at 1 h and 24 h [79] was observed. Nine RCTs used a 30 mg/kg bolus; a 500 mg/h infusion [76] for 24 h showed a pain reduction at 15 and 30 min in 40 subjects; an infusion of 9 mg/kg/h in 294 subjects (for 1 h) [74] showed a significant decrease in pain; a 10 mg/kg/h infusion [69,71] for 24 h showed a pain reduction at 12 h in 70 subjects, and at 24 h in 50 subjects; and 20 mg/kg/h [68] showed a decrease of pain for 24 h in 80 subjects.

With a bolus of 40 mg/kg followed by an infusion of 10 mg/kg/h over 24 h, a decrease of pain at 24 h was observed [67]. Ten RCTs reported a pain decrease for a bolus of 50 mg/kg followed by an infusion ranging from 500 mg/h to 25 mg/kg/h [49,51,52,53,54,55,58,59,63,64] and from 24 h [49,52,53,54,58,59,63,64] to 48 h [51,52,55]. Four RCTs showed a significant decrease in pain at 24 h [53,54,58,64], 2 RCTs at 12 h [49,59] and 1 RCT at 30 min [63]. Two RCTs showed a pain decrease between 0 to 48 h [51,55] and one RCT from 4 to 48 h [52].

A number of RCTs (16) did not show however any efficacy of Mg on pain [36,38,42,44,50,56,57,61,62,66,70,72,73,75,77,81]. Six intravenous Mg sulphate regimens ranging from non-bolus infusion to 50 mg/kg bolus followed by infusion showed no efficacy on pain. Concerning RCTs without bolus administration, four RCTs were negative with an infusion of 5 mg/kg during surgery in 60 subjects [36], with an infusion of 50 mg/kg during surgery in 50 subjects [44] and 75 subjects [38], and with an infusion of 150 mg/kg during surgery [42]. Concerning bolus administration of 50 mg/kg, five RCTs showed no efficacy on reduction of post-operative pain: two RCTs with an infusion of 8 mg/kg/h in 46 [62] and 60 subjects [61], one RCT with an infusion of 10 mg/kg/h for 24 h in 40 subjects [57] and two RCTs with an infusion of 15 mg/kg/h in 58 subjects [50] and 62 subjects [56] over 24 and 72 h. With a 40 mg/kg bolus followed by a 10 mg/kg/h infusion, no pain improvement was observed over 24 h in 40 subjects [66]. Furthermore, four RCTs did not show any difference in the progression of pain: for a bolus administration of 30 mg/kg of Mg sulphate followed by an infusion of 6 mg/kg/h over 120 min in 42 subjects [75], or by an infusion of 10 mg/kg/h in 84, 96 or 100 subjects [70,72,73]. Another RCT did not show efficacy in reducing pain over 5 days of 20 mg/kg Mg sulphate bolus followed by an infusion of 10 mg/kg/30 min during surgery in 24 subjects [77]. Finally, a 4 g bolus in 200 subjects gave negative results [81] (Table 1).

In renal pain, 5 double-blind RCTs [82,83,84,85,86] included patients receiving an infusion of 15 mg/kg of Mg sulphate versus NSAIDs (Non-steroidal anti-inflammatory) [84] or versus a reference treatment (0.1 mg/kg of morphine + 30 mg of ketorolac [85]); patients receiving 50 mg/kg Mg sulphate during surgery [82] or 50 mg/kg Mg sulphate during 30 min [83]; and those receiving 2 cc of Mg sulphate during 15 min [86] (*n* = 453). Among these studies, two RCTs showed the efficacy of Mg on pain reduction using VAS at 30 and 60 min [84,85] (Table 1).

In migraine, 18 RCTs explored pain evolution with Mg: nine RCTs studied the effectiveness of the intravenous (IV) Mg sulphate [87,88,89,90,91,92,93,94,95] and nine RCTs studied the effectiveness of oral Mg on headaches [98,99,100,101,102,103,104,105,106] (*n* = 1248). Nine RCTs studied the effects of IV Mg sulphate on pain reduction in migraine (*n* = 622). Four RCTs showed a positive effect of Mg in reducing pain. A significant reduction in pain after 2 h (VAS baseline: 8; VAS 2 h: 0) in 70 subjects following IV administration of 2 g Mg sulphate versus 60 mg IV caffeine [87] was observed. Shahrami et al. showed a significant pain reduction over 2 h after IV administration of Mg sulphate dosed at 1 g in 100 mL saline, in 70 subjects (VAS baseline: 8/10; VAS at 2 h: 0.66) [92]. A significant pain diminution for subjects with migraine and aura at 1 h following administration of 1 g Mg sulphate in 60 subjects (VAS Mg: 4/10; placebo: 6/10) [95] was observed. Demirkaya et al. showed using a qualitative pain scale the beneficial effects of IV supplementation of 1 g Mg sulphate versus placebo in 30 subjects over 2 h [93]. Five RCTs did not show a decrease in pain following IV Mg sulphate administration in migraine headaches [88,89,90,91,94]. No reduction in pain was obtained following administration of 2 g IV Mg sulphate in 42 subjects [83]. Ginder et al. showed no significant effect of 2 g Mg sulphate IV in 36 subjects over 4 h [90]. Corbo et al. did not show any beneficial effect of 2 g IV Mg sulphate associated with metoclopramide versus metoclopramide and placebo in 44 subjects over a 24-month follow-up (mean change VAS from baseline to final: VAS Mg: 55/100 ± 32 versus VAS Placebo: 71/100 ± 27) [94]. Cete et al. showed no significant effect of 2 g Mg sulphate on pain reduction measured with VAS at 0, 15 and 30 min in 113 subjects [91]. Furthermore, Kandil et al. reported no significant difference in pain reduction between magnesium, metoclopramide and prochlorperazine [88].

**Figure 2:**
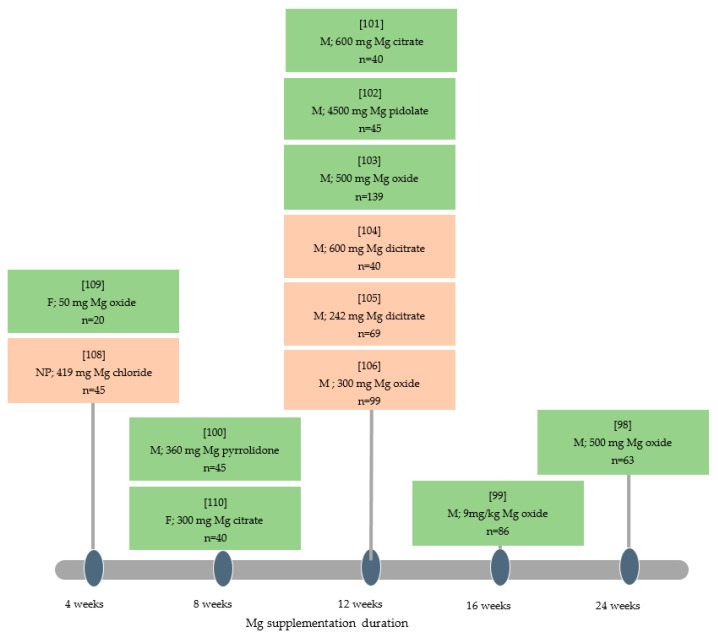
Oral magnesium in several pain situations. In green, significant reduction of pain; in orange: no significant improvement of pain. NP: Neuropathic Pain; F: Fibromyalgia; M: Migraine.

Concerning the nine RCTs evaluating the effect of oral magnesium on headache (*n* = 626), three double-blind RCTs in crossover [98] or parallel arms [99,100] (*n* = 148) showed a reduction in migraine pain intensity as measured by the Total Bread Index (TBI) (−55 points at 2 months) [99], Headache Impact Test-6 (HIT-6) (−16 points at 24 weeks) [98], and VAS (−3.57 at 12 weeks and −4.5 at 24 weeks) [99,100]. These studies used 500 mg of Mg oxide over 24 weeks [98], 360 mg of Mg pyrrolidone carboxylic acid over 2 months [100] and 600 mg of Mg citrate over 3 months [99]. Five double-blind RCTs (out of 9) in crossover [98], parallel arms [99,100,101] or open label design [102] (*n* = 279) showed a decrease in migraine frequency in the Mg-treated groups (−4.4 ± 1.7 days on 6 months) [98,99,100,101,102] for 360 mg of Mg pyrrolidone carboxylic acid over 2 months [100], 4500 mg of Mg pidolate [102] and 600 mg of Mg citrate [99] over 3 months, 9 mg/kg of Mg oxide over 16 weeks [101] and 500 mg of Mg oxide over 24 weeks [98]. A single-blind clinical trial in parallel groups showed a significant reduction in migraine frequency per day with magnesium oxide 500 mg supplementation in 139 subjects over 12 weeks [103]. Two double-blind RCTs (out of 9) in crossover [98] or parallel arms [99] (*n* = 103) showed a decrease in migraine duration in the Mg group from 49 h to 16 h over 24 weeks [98,99] with 600 mg of Mg citrate over 3 months [99] and 500 mg of Mg oxide over 24 weeks [98]. Three RCTs (out of 9) gave negative results on the effect of oral magnesium on headaches [104,105,106] (*n* = 208). Among these three RCTs, two did not show any significant effect of Mg in reducing pain at 12 weeks [104,105] (*n* = 109) with a supplementation of 600 mg of Mg dicitrate over 12 weeks [104] and 242 mg of Mg u-aspartate-hydrochloride-trihydrate over 12 weeks [105]. Maizels et al. showed no effect on the number of migraine days following magnesium oxide 300 mg supplementation in 99 subjects over 3 months [106] (Figure 2).

In chronic pain (Neuropathic Pain and Complex Regional Pain Syndrome (CRPS)), six double-blind RCTs in crossover [15,96] or parallel arms [18,97,107,108] studied the impact of Mg (*n* = 232). They used different Mg dosages (30 mg/kg Mg sulphate for 30 min [96]; bolus of 0,16 mmol/kg of Mg chloride followed by an infusion of 0,16 mmol/kg/h of Mg chloride [18]; 1 g Mg sulphate in 250 mL saline 0.9% over 4 h every day for 2 week and then 400 mg Mg oxide + 100 mg Mg gluconate orally twice daily during 4 weeks [97]; 6 × 419 mg Mg chloride per day for one month [108]; 0.5 mg/kg ketamine + 3 g of Mg sulphate over 30 min once [15] and 1000 mg of intramuscular Mg sulphate in week 1, 1500 mg in week 2 and 2000 mg in week 3 [107]). Two studies showed a reduction of pain using VAS at 20, 30 min [96] and at 6 weeks [97] after intravenous [96,97] or oral [97] administration of Mg (*n* = 87). Four studies did not show any pain reduction [15,18,107,108] (*n* = 145).

In fibromyalgia, 2 RCTs explored the effectiveness of oral magnesium (*n* = 60) [109,110]: the first RCT was a randomized, double-blind, placebo-controlled, crossover study for a 2-month period with a low fixed dosage, and subsequent trial was a 6-month open-label, escalated dosage trial. The patients took three tablets of Mg malate twice daily and increased their dosage every 3–5 days until they experienced acceptable outcomes or related side effects for a 6-month period (*n* = 20) [109]. In the second RCT, 3 parallel groups of treatment have been compared: Magnesium citrate (300 mg/day), amitriptyline (10 mg/day) and amitriptyline (10 mg/day) + magnesium citrate (300 mg/day) for 8 weeks. This trial showed a reduction of the number of tender points (from 15.2 to 11.7 points), the tender point index (from 27 to 19.4 points), depression level (from 12.9 to 8 points) and fibromyalgia impact questionnaire score (from 35.4 to 23.6 points) with Mg treatment for 8 weeks (*n* = 40) [110] (Figure 2).

### 3.2. Magnesium and Analgesics Consumption

Concerning the impact of Mg on analgesics consumption, 45 RCTs out of 49 in post-operative pain reported the analgesics required during the study after surgery [33,34,35,36,37,38,39,40,41,42,44,45,46,47,48,49,50,51,52,54,55,56,57,58,59,60,61,62,64,65,66,67,68,69,70,71,73,74,75,76,77,78,79,80,81], (*n* = 3146); 36/45 RCTs showed a significant decrease in analgesics consumption in post-operative pain after Mg treatment compared to placebo or conventional treatment group with a large panel of drugs: (morphine [35,41,42,49,51,52,54,56,57,58,61,64,65,66,67,68,69,70,76,77,78,79,81] fentanyl [44,46,50,62,73,75,81], tramadol [36,38,39,40,46,47,73,80] pethidine [34,45,59,71,74] diclofenac [33,36,81], desflurane [67], piritramide [37], metamizol [36], propofol [48,67] and ketorolac [46,51,52]). However, 11 RCTs showed no significant difference in analgesics consumption after treatment with Mg (morphine [54,57,66,70,77,81] fentanyl [50], tramadol [36,47,73], coproxomal [81] and meperidine [63]) (Table 1). In migraine, two RCTs showed the impact of Mg supplementation on the reduction of analgesics consumption [91,95] (*n* = 173). In renal colic, only one RCT reported a significant decrease of morphine consumption in the Mg group compared to placebo [85].

### 3.3. Bioavailability of Magnesium Salts

This review explored also publications on the bioavailability of the different Mg salts in order to identify specificities among pharmaceutical preparations. A number of publications have studied 17 Mg salts in preclinical and clinical conditions [111,112,113,114,115,116,117,118]. Comparison of the oral bioavailability and absorption of different pharmaceutical forms of inorganic and organic Mg salts has been explored in 5 RCTs [113,114,115,116,117] (Table 2). These publications show that Mg citrate is more bioavailable than Mg oxide [113,114], and that Mg oxide or chloride with a specific matrix [115,116] or when combined with other salts has a better bioavailability [117]. Apart from these 5 RCTs, there is, however, no large scale trial comparing organic and inorganic salts. Furthermore, no study compared head to head Mg pharmaceutical forms on efficacy and safety in pain conditions.

## 4. Discussion

This literature review aimed at evaluating how Mg may relieve pain. Pain is a complex phenomenon and different types of pain have been described, including somatic, complex, or psychogenic, idiopathic, or acute, chronic, or nociceptive, neuropathic and nociplastic [119]. All types of pain have been selected in this review according to an adequate RCT methodology. Hence, RCTs on somatic (post-operative pain) and complex pain (renal colic, chronic pain, migraine and fibromyalgia) have been explored. The number of RCTs amounts only to 81, 45 RCTs for effectiveness on pain and 40 for analgesics diminution. Collective results show a modest effect of Mg in a majority of studies and this review stresses a number of gaps.

First, there is a large heterogeneity concerning the methodologies used in the different trials. Different pathologies, missing information on patients disease, different Mg chemical forms or different settings have been chosen. Several routes of administration have been used, intravenous or oral intake, making comparisons difficult. Several pathologies have been treated and evaluation tools differed between studies and across the same pathology. A total of 8 chemical forms out of the 17 forms tested for bioavailability have been used and numerous intravenous (28) and oral (11) Mg dosages have been administered.

Concerning the management of post-operative pain, no universal dosage has been defined for the use of Mg sulphate. The 39 different Mg dosages used for pain alleviation in the RCTs of this review are far above the dosage of Mg sulphate commonly used in current practice (the most frequent in RCTs being 30 mg/kg bolus followed by an infusion of 10 mg/kg/hour). The wide variety of dosage regimens in the administration of Mg sulphate in post-operative pain and the controversial results in terms of its effectiveness in reducing pain (66% of RCTs show a reduction in pain) and in the consumption of analgesics (73% of RCTs show a reduction in analgesics) do not allow us to draw conclusions about a universal reference dosage for this indication. Clinical trials aimed at defining an optimal dosage of Mg sulphate in post-operative pain are needed.

In post-surgery, opioids are largely used and have adverse effects well described in the literature such as nausea, vomiting, constipation and addiction [120]. In addition to these adverse events, paradoxical hyperalgesia may be triggered and NMDAR antagonists may have a beneficial role to play in this situation [121]. A decrease in analgesics consumption with Mg-based treatment in post-operative pain has been observed in many trials. Mg appears to be a good non-drug alternative for reducing post-operative pain by limiting the side-effects of commonly prescribed opioids, but this aspect needs to be explored further.

A recurrent question concerns the best choice of Mg pharmaceutical form and Mg dosage for pain alleviation. While Mg sulphate is commonly used intravenously, bioavailability studies recommend the use of second- (e.g., gluconate, citrate, lactate, pidolate, L-aspartate) and third-generation (e.g., glycerophosphate and bisglycinate) Mg salts compared to the first generation (e.g., carbonate, chloride and oxide), but RCTs vary in Mg dosages and duration, making it difficult to identify a reference salt and an optimal duration of Mg supplementation.

In the case of other pathologies, 44% of RCTs in migraine, 40% in renal pain and 50% in chronic pain observed significant reductions in pain following Mg treatment. Despite encouraging results in migraine and renal colic pain, RCTs exploring the efficacy of Mg on such different pain situations are still seldom. Moreover, the use of different dosages and treatment durations of oral Mg in migraine and of intravenous Mg in renal colic pain do not allow us to conclude on a reference dosage. Additional clinical trials are needed to support the efficacy of Mg in these types of pain.

The Food and Drug Administration (FDA) recommends a daily oral Mg intake of around 400 mg for a man and 310 mg for a woman between 19 and 30 years old [122]. Oral Mg supplements used in the management of pain such as migraine, fibromyalgia, chronic pain and neuropathic pain are in accordance with minimum FDA recommendations with the exception of 2 RCTs [105,109]. Indeed, while studies on the bioavailability of the different generations of Mg salts recommend second and third generation salts, results show the good efficacy of Mg oxide [98,99,101,109] but failure in pain alleviation with second generation salts [104,105]. Our review on bioavailability, focused on RCTs, identified 5 RCTs that stress the superiority of Mg citrate or Mg oxide with novel matrices. The superiority of a pharmaceutical form would need further studies, as there are no head to head studies evaluating the efficacy of Mg. All the more that improved bioavailability does not allow to extrapolate to an improved efficacy unless it is demonstrated. For oral administration, as for intravenous administration, there are contested results, and only 9 RCTs with 7 different pharmaceutical forms. Clinical trials testing different generations of Mg salts at recommended FDA dosages are necessary to determine whether differences in efficacy occur depending on the generation used.

There are also gaps in the literature concerning the use of Mg in major public health diseases that commonly generate pain. More information is needed on cancer pain, fibromyalgia, CRPS and rheumatic diseases. For example, osteoporosis mainly affects postmenopausal women and concerns 40 millions in the USA [123]. It is a pathology that reduces bone mass, resulting in an increased risk of fracture frequently associated with significant pain and suffering [124]. In addition, the literature describes a link between low plasma Mg levels and the onset of osteoporosis [125]. Due to its involvement in tissue structure at the level of hydroxyapatite crystals, Mg seems to be effective in reducing the onset of osteoporosis [126]. To date, there is no randomised clinical trial evaluating the link between Mg supplementation and osteoporosis in the literature.

Another observation is that Mg may have different modes of action in acute and chronic pain situations. LTP follows two stages after a nociceptive stimulus: an initial and a later stage, and the NMDAR is fully involved [127] as a modulator of LTP development [128]. The inhibition of NMDAR is one of the keys to blocking LTP. NMDAR antagonists like Mg may not only cure but also prevent the development of central sensitization [129,130], a very important aspect of 4P Medicine. However, available RCTs do not include a long follow-up period after surgery to identify if Mg could have an impact on the prevention of central sensitization. This needs to be addressed in future studies.

Finally, there is also a need to focus on comorbidities that always accompany pain, like fatigue, stress, anxiety and depression. Studies conducted on the use of Mg in chronic stress have shown satisfactory results [5]. Stressed subjects show a decrease in their level of stress following various oral supplements (192 mg Mg lactate over 3 and 6 weeks [7]; 300 mg Mg oxide over 4 and 8 weeks [11] and 75 mg Mg over 12 weeks [131]). In addition, Mg supplementation of 400 mg promotes a better physiological regulation of sympathetic and parasympathetic efferent as measured by a heart rate variability test in stressed subjects [132]. Several ongoing clinical trials tackle pain alleviation by addressing the impact of Mg on stress in painful patients. An ongoing RCT, *Semafor* (NCT0388700) explores stress, pain and sleep in fibromyalgia patients, focusing on the central role of Mg in the vicious circle of stress and pain [5]. Another ongoing RCT, *Magritte* (NCT04391452), is studying the impact of a Mg-based dietary supplement on stress, pain and comorbidities with a double approach, clinical and mechanistic with functional magnetic resonance imaging (fMRI) neuroimaging. These studies aim at deciphering the ubiquitous mode of action of Mg in pain and stress, and its pivotal position in improving, beyond pain, the quality of life of vulnerable patients.

## 5. Conclusions

Collective data on the management of pain with Mg are modest and controversial, and underline the need for recommendations on Mg dosages in post-surgery, in chronic pain, intravenously or orally, for patients in hospital or in the community wishing to start supplementation. Additional clinical trials are needed to achieve a sufficient level of evidence about the efficacy of the different available Mg pharmaceutical forms. Beyond pain, Mg with its physiological NMDAR antagonism, with its pivotal place as a mediator in pain comorbidities, and complex mechanism of action, appears as a valuable non-drug approach to be explored further in order to optimise the quality of life of patients in pain.

## Figures and Tables

**Table 1:** Randomised clinical trials evaluating the effect of intravenous magnesium sulphate on pain and analgesics consumption compared to controls in different pain situations. Studies are versus (vs.) placebo, double-blind and in parallel groups unless specified. “ND”: not determined; * not double-blind; CrO: cross-over. The bolus corresponds to the first post-operative injection, followed by an infusion according to the protocol. Negative studies are with a grey highlight.

Indications	Authors	*n*	Mg		Pain Diminution	Analgesics Consumption Diminution
			Bolus	Infusion		
Post-surgery Pain	[33]	100	/	30 mg/kg *	*p* < 0.05	*p* < 0.05
	[34]	40	/	15 mg/kg/h	*p* = 0.0001	*p* = 0.0001
	[35]	60	/	8 mg/kg/h	*p* < 0.01	*p* < 0.01
	[36]	60	/	7.5 mg/kg *	*p* < 0.05	*p* < 0.001
			/	5 mg/kg	*p >* 0.05	*p* > 0.05
	[37]	24	/	50 mg/kg–30 min	*p* < 0.05	*p* < 0.05
	[38]	75	/	50 mg/kg–30 min *	*p >* 0.05	*p* < 0.05
	[39]	70	/	50 mg/kg–30 min	ND	*p* < 0.001
	[40]	30	/	50 mg/kg–20 min	ND	*p* < 0.001
	[41]	50	/	50 mg/kg–15 min	*p* < 0.05	*p* < 0.001
	[42]	60	/	150 mg *	*p >* 0.05	*p* > 0.05
	[43]	38	/	65 mg/kg	*p* < 0.001	ND
	[44]	50	/	50 mg/kg	*p* > 0.05	*p* < 0.01
	[45]	40	/	50 mg/kg	*p* < 0.05	*p* = 0.0001
	[46]	57	/	50 mg/kg	*p* = 0.034	*p* = 0.043
	[47]	83	/	50 mg/kg	*p* < 0.05	*p* > 0.05
	[48]	120	/	30 mg/kg	ND	*p* < 0.001
	[49]	120	50 mg/kg	25 mg/kg/h	*p* < 0.05	*p* < 0.001
	[50]	58	50 mg/kg	15 mg/kg/h	*p* > 0.05	*p* > 0.05
	[51]	40	50 mg/kg	15 mg/kg/h *	*p* < 0.001	*p* < 0.001
	[52]	50	50 mg/kg	15 mg/kg/h	*p* = 0.011	*p* = 0.005
	[53]	74	50 mg/kg	15 mg/kg/h	*p* = 0.009	ND
	[54]	50	50 mg/kg	15 mg/kg/h	*p* < 0.05	*p* = 0.07
	[55]	44	50 mg/kg	15 mg/kg/h	*p* = 0.001	*p* = 0.014
	[56]	62	50 mg/kg	15 mg/kg/h	*p* > 0.05	*p* = 0.042
	[57]	40	50 mg/kg	10 mg/kg/h	*p* > 0.05	*p* > 0.05
	[58]	60	50 mg/kg	10 mg/kg/h	*p* < 0.05	*p* < 0.006
	[59]	30	50 mg/kg	8 mg/kg/h	*p* < 0.0001	*p* < 0.05
	[60]	120	50 mg/kg	8 mg/kg/h	ND	*p* < 0.05
	[61]	60	50 mg/kg	8 mg/kg/h	*p* > 0.05	*p* < 0.05
	[62]	46	50 mg/kg	8 mg/kg/h	*p* > 0.05	*p* < 0.05
	[63]	50	50 mg/kg	8 mg/kg/h	*p* < 0.05	ND
	[64]	48	50 mg/kg	500 mg/h	*p* < 0.05	*p* = 0.0002
	[65]	80	40 mg/kg	20 mg/k/h	ND	*p* < 0.001
				10 mg/kg/h	ND	*p* < 0.001
	[66]	40	40 mg/kg	10 mg/kg/h	*p* > 0.05	*p* = 0.52
	[67]	60	40 mg/kg	10 mg/kg/h	*p* = 0.024	*p* = 0.048
	[68]	80	30 mg/kg	20 mg/kg/24 h	*p* = 0.001	*p* = 0.001
	[69]	50	30 mg/kg	10 mg/kg/h *	*p* < 0.05	*p* < 0.05
	[70]	96	30 mg/kg	10 mg/kg/h	*p* > 0.05	*p* > 0.05
	[71]	70	30 mg/kg	10 mg/kg/h	*p* < 0.001	*p* < 0.001
	[72]	100	30 mg/kg	10 mg/kg/h	*p* = 0.29	ND
	[73]	84	30 mg/kg	10 mg/kg/h	*p* > 0.05	*p* > 0.05
	[74]	294	30 mg/kg	9 mg/kg/h	*p* < 0.0001	*p* < 0.0001
	[75]	42	30 mg/kg	6 mg/kg/h	*p* > 0.05	*p* < 0.05
	[76]	40	30 mg/kg	500 mg/h	*p* < 0.05	*p* < 0.05
	[77]	45	20 mg/kg	10 mg/kg–30 min * vs. fentanyl and ketamine	*p* > 0.05	*p* > 0.05
	[78]	74	20 mg/kg	20 mg/kg/h	*p* = 0.005	*p* = 0.001
	[79]	36	20 mg/kg	2 mg/kg/h	*p* < 0.01	*p* = 0.001
	[80]	108	250 mg	20 mg/kg/h	*p* = 0.001	*p* = 0.033
	[81]	200	4 g	/	*p* > 0.05	*p* > 0.05
Renal Pain	[82]	87	/	50 mg/kg	*p* = 0.232	ND
	[83]	80	/	50 mg/kg–20 min vs. morphine	*p* > 0.05	ND
	[84]	96	/	15 mg/kg–15 min vs standard treatment	*p* < 0.05	ND
	[85]	100	/	15 mg/kg–15 min	*p* = 0.001	*p* = 0.043
	[86]	90	/	2 cc–15 min vs morphine	*p* = 0.799	ND
Migraine	[87]	70	/	2 g–10 min * vs. caffeine	*p* < 0.05	ND
	[88]	157	/	2 g–20 min vs. prochlorperazine/ metoclopramide	*p* > 0.05	*p* > 0.05
	[89]	42	/	2 g–10 min	*p* = 0.63	ND
	[90]	36	/	2 g–10 min vs. prochlorperazine	*p* > 0.05	*p* > 0.05
	[91]	113	/	2 g–10 min	*p* > 0.05	*p* < 0.05
	[92]	70	/	1 g–15 min vs. dexamethasone/ metoclopramide	*p* < 0.0001	ND
	[93]	30	/	1 g–15 min *	*p* < 0.0001	ND
	[94]	44	2 g	/	*p* > 0.05	*p* > 0.05
	[95]	60	1 g	/	*p* < 0.05	*p* < 0.05
Chronic Pain	[96]	7	/	30 mg/kg–30 min; CrO	*p* = 0.016	ND
	[15]	60	/	3 g–30 min; CrO	*p* = 0.296	ND
	[97]	80	/	1 g–4 h	*p* = 0.034	ND
	[18]	10	0.16 mmol/kg	0.16 mmol/kg/h	*p* = 0.084	ND

**Table 2:** Magnesium bioavailability in randomised clinical trials comparing inorganic and organic salts in healthy volunteers (*n* = number). DB: double-blind; P: parallel; CrO: cross-over.

Authors	*n*	Type of Study	Inorganic Mg Salts	Organic Mg Salts	Combination of Mg Salts	Conclusions
[113]	17	P	Mg oxide (60% Mg element: 15 mmol)	Mg citrate (16% Mg element: 4 mmol)	/	Mg citrate is more soluble than Mg Oxide in water (55% vs. 0.8%, *p* < 0.05), less ph-dependent with lesser ionic concentrations.
[114]	46	DBP	Mg oxide (60% Mg element: 180 mg)	Mg citrate (16% Mg element: 48 mg); Mg amino-acid chelate: 300 mg (% Mg element: ND)	/	Mg citrate then amino-acid chelate are more bioavailable than Mg oxide (*p* < 0.02).
[115]	10	DBCrO	Mg oxide (60% Mg element: 210 mg)/Mg oxide with a sucrester matrix (210 mg)	Mg citrate (16% mg element: 56 mg); Mg bisglycinate (20% Mg element: 70 mg)	/	Mg oxide with a sucrester matrix has a higher Mg bioavailability (*p* < 0.05).
[117]	20	DBCrO	Mg oxide (60% Mg element: 241.3, 300, 400, 450, 500 mg); Mg carbonate (40% Mg element: 100 mg); Mg chloride (12% Mg element: 71.5 mg)	Mg citrate (16% Mg element: 19 mg; 100 and 200 mg)	Mg oxide (60% Mg element: 149 mg) + glycerophosphate (12.37% Mg element: 47 mg); Mg citrate (16% Mg element) + Mg bis hydrogen-L-glutamate (Mg element: ND): 40 mg; Mg orotate dihydrate: 32.8 mg (% mg element: ND); Mg glycinate lysinate chelate (20% Mg element: 100 mg)	Higher bioavailability when Mg oxide is combined (*p* < 0.005)
[116]	20	CrO	Mg chloride with a novel matrix: 100 mg Mg element) vs. Mg carbonate (3 × 100 mg Mg element)	/	/	Mg chloride with a novel matrix has a better bioavailability

## References

[1] Garland E.L. (2012). Pain Processing in the Human Nervous System. Prim. Care Clin. Off. Pract..

[2] Martucci K.T., Mackey S.C. (2018). Neuroimaging of Pain. Anesthesioogy.

[3] Loeser J.D., Melzack R. (1999). Pain: An overview. Lancet.

[4] Jank R., Gallee A., Boeckle M., Fiegl S., Pieh C. (2017). Chronic Pain and Sleep Disorders in Primary Care. Pain Res. Treat..

[5] Pickering G., Mazur A., Trousselard M., Bienkowski P., Yaltsewa N., Amessou M., Noah L., Pouteau E. (2020). Magnesium Status and Stress: The Vicious Circle Concept Revisited. Nutrients.

[6] Silberstein S., Loder E., Diamond S., Reed M.L., Bigal E.M., Lipton R.B. (2007). Probable Migraine in the United States: Results of The American Migraine Prevalence and Prevention (AMPP) Study. Cephalalgia.

[7] Boyle N.B., Lawton C., Dye L. (2017). The Effects of Magnesium Supplementation on Subjective Anxiety and Stress—A Systematic Review. Nutrients.

[8] Fuentes J.C., Salmon A.A., Silver M.A. (2006). Acute and Chronic Oral Magnesium Supplementation: Effects on Endothelial Function, Exercise Capacity, and Quality of Life in Patients with Symptomatic Heart Failure. Congest. Hear. Fail..

[9] Magnesium Saw Huge Cross-Channel Sales Growth Last Year. Here’s What’s Driving the Ingredient in 2020: 2020 Ingredient Trends to Watch for Foods, Drinks, and Dietary Supplements. https://www.nutritionaloutlook.com/view/magnesium-saw-huge-cross-channel-sales-growth-last-year-heres-whats-driving-ingredient.

[10] Kirkland A.E., Sarlo G.L., Holton K.F. (2018). The Role of Magnesium in Neurological Disorders. Nutrients.

[11] Pouteau E., Kabir-Ahmadi M., Noah L., Mazur A., Dye L., Hellhammer J., Pickering G., DuBray C. (2018). Superiority of magnesium and vitamin B6 over magnesium alone on severe stress in healthy adults with low magnesemia: A randomized, single-blind clinical trial. PLoS ONE.

[12] Schwalfenberg G.K., Genuis S.J. (2017). The Importance of Magnesium in Clinical Healthcare. Scientifica.

[13] Ng K.T., Yap J.L., Izham I.N., Teoh W.Y., Kwok P.E., Koh W.J. (2020). The effect of intravenous magnesium on postoperative morphine consumption in noncardiac surgery. Eur. J. Anaesthesiol..

[14] Nadeson R., Tucker A., Bajunaki E., Goodchild C. (2002). Potentiation by ketamine of fentanyl antinociception. I. An experimental study in rats showing that ketamine administered by non-spinal routes targets spinal cord antinociceptive systems. Br. J. Anaesth..

[15] Pickering G., Pereira B., Morel V., Corriger A., Giron F., Marcaillou F., Bidar-Beauvallot A., Chandeze E., Lambert C., Bernard L. (2020). Ketamine and Magnesium for Refractory Neuropathic Pain. Anesthesiology.

[16] Duncan W.C., Zarate C.A. (2013). Ketamine, Sleep, and Depression: Current Status and New Questions. Curr. Psychiatry Rep..

[17] Corriger A., Pickering G. (2019). Ketamine and depression: A narrative review. Drug Des. Dev. Ther..

[18] Felsby S., Nielsen J., Arendt-Nielsen L., Jensen T.S. (1996). NMDA receptor blockade in chronic neuropathic pain: A comparison of ketamine and magnesium chloride. Pain.

[19] Nikolaev M.V., Magazanik L.G., Tikhonov D.B. (2012). Influence of external magnesium ions on the NMDA receptor channel block by different types of organic cations. Neuropharmacology.

[20] Li X.-H., Miao H.-H., Zhuo M. (2019). NMDA Receptor Dependent Long-term Potentiation in Chronic Pain. Neurochem. Res..

[21] Blanke M.L., Van Dongen A.M.J., Van Dongen A.M. (2009). Activation Mechanisms of the NMDA Receptor. Biology of the NMDA Receptor.

[22] Gambrill A.C., Storey G.P., Barria A. (2011). Dynamic Regulation of NMDA Receptor Transmission. J. Neurophysiol..

[23] Fukunaga K., Muller D., Miyamoto E. (1996). CaM Kinase II in Long-Term Potentiation. Neurochem. Int..

[24] Ives NMDA Receptors Play Key Role in Sleep Deficits that Accompany Psychiatric Disorders. News-Medicalnet 2020. https://www.news-medical.net/news/20200605/NMDA-receptors-play-key-role-in-sleep-deficits-that-accompany-psychiatric-disorders.aspx.

[25] Barkus C., McHugh S.B., Sprengel R., Seeburg P.H., Rawlins J.N.P., Bannerman D.M. (2010). Hippocampal NMDA receptors and anxiety: At the interface between cognition and emotion. Eur. J. Pharmacol..

[26] Chen W., Liu S., Chen F., Zhou C., Zhuang C., Shao S., Yu J., Huang D., Chen B., Yu Z. (2015). Relationship between NMDA receptor and postoperative fatigue syndrome and its associated central mechanism. Chin. J. Gastrointest. Surg..

[27] Chen L.-F., Yang C.-H., Lin T.-Y., Pao P.-J., Chu K.C.-W., Hsu C.-W., Bai C.-H., Du M.-H., Hsu Y.-P. (2020). Effect of magnesium sulfate on renal colic pain. Medicine.

[28] Maier J.A., Pickering G., Giacomoni E., Cazzaniga A., Pellegrino P. (2020). Headaches and Magnesium: Mechanisms, Bioavailability, Therapeutic Efficacy and Potential Advantage of Magnesium Pidolate. Nutrients.

[29] Park R., Ho A.M.-H., Pickering G., Arendt-Nielsen L., Mohiuddin M., Gilron I. (2020). Efficacy and Safety of Magnesium for the Management of Chronic Pain in Adults: A Systematic Review. Anesthesia Analg..

[30] Choi H., Parmar N. (2013). The use of intravenous magnesium sulphate for acute migraine. Eur. J. Emerg. Med..

[31] Chiu H.-Y., Yeh T.-H., Huang Y.-C., Chen P.-Y. (2016). Effects of Intravenous and Oral Magnesium on Reducing Migraine: A Meta-analysis of Randomized Controlled Trials. Pain Physician.

[32] Shi L., Zhu H., Ma J., Shi L.L., Gao F., Sun W. (2021). Intra-articular magnesium to alleviate postoperative pain after arthroscopic knee surgery: A meta-analysis of randomized controlled trials. J. Orthop. Surg. Res..

[33] Kiran S., Gupta R., Verma D. (2011). Evaluation of a single-dose of intravenous magnesium sulphate for prevention of postoperative pain after inguinal surgery. Indian J. Anaesth..

[34] Haryalchi K., Abedinzade M., Khanaki K., Ghanaie M.M., Zadeh F.M. (2017). Whether preventive low dose magnesium sulphate infusion has an influence on postoperative pain perception and the level of serum beta-endorphin throughout the total abdominal hysterectomy. Rev. Esp. Anestesiol. Reanim. Engl. Ed..

[35] Dabbagh A., Elyasi H., Razavi S.S., Fathi M., Rajaei S. (2009). Intravenous magnesium sulfate for post-operative pain in patients undergoing lower limb orthopedic surgery. Acta Anaesthesiol. Scand..

[36] Kocman I.B., Krobot R., Premuzić J., Kocman I., Stare R., Katalinić L., Basić-Jukić N. (2013). The effect of preemptive intravenous low-dose magnesium sulfate on early postoperative pain after laparoscopic cholecystectomy. Acta Clin. Croat..

[37] Levaux C., Bonhomme V., Dewandre P.Y., Brichant J.F., Hans P. (2003). Effect of intra-operative magnesium sulphate on pain relief and patient comfort after major lumbar orthopaedic surgery. Anaesthesia.

[38] Demiroglu M., Ün C., Ornek D.H., Kıcı O., Yıldırım A.E., Horasanlı E., Başkan S., Fikir E., Gamli M., Dikmen B. (2016). The Effect of Systemic and Regional Use of Magnesium Sulfate on Postoperative Tramadol Consumption in Lumbar Disc Surgery. BioMed Res. Int..

[39] Gucyetmez B., Atalan H., Aslan S., Yazar S., Polat K. (2016). Effects of Intraoperative Magnesium Sulfate Administration on Postoperative Tramadol Requirement in Liver Transplantation: A Prospective, Double-Blind Study. Transplant. Proc..

[40] Tauzin-Fin P., Sesay M., Delort-Laval S., Krol-Houdek M.C., Maurette P. (2006). Intravenous magnesium sulphate decreases postoperative tramadol requirement after radical prostatectomy*. Eur. J. Anaesthesiol..

[41] Mireskandari S.M., Pestei K., Hajipour A., Jafarzadeh A., Samadi S., Nabavian O. (2015). Effects of Preoperative Magnesium Sulphate on Post-Cesarean Pain, A Placebo Controlled Double Blind Study. J. Fam. Reprod. Health.

[42] Arora M.K., Muthiah T., Trikha A., Sunder A.R., Prasad G., Singh P.M. (2016). Efficacy of magnesium as an adjuvant to bupivacaine in 3-in-1 nerve block for arthroscopic anterior cruciate ligament repair. Indian J. Anaesth..

[43] Kahraman F., Eroglu A. (2014). The Effect of Intravenous Magnesium Sulfate Infusion on Sensory Spinal Block and Postoperative Pain Score in Abdominal Hysterectomy. BioMed Res. Int..

[44] Schulz-Stubner S., Wettmann G., Reyle-Hahn S.M., Rossaint R. (2001). Magnesium as part of balanced general anaesthesia with propofol, remifentanil and mivacurium: A double-blind, randomized prospective study in 50 patients. Eur. J. Anaesthesiol..

[45] Taheri A., Haryalchi K., Ghanaie M.M., Arejan N.H. (2015). Effect of Low-Dose (Single-Dose) Magnesium Sulfate on Postoperative Analgesia in Hysterectomy Patients Receiving Balanced General Anesthesia. Anesthesiol. Res. Pract..

[46] Kim J.E., Shin C.S., Lee Y.C., Lee H.S., Ban M., Kim S.Y. (2015). Beneficial effect of intravenous magnesium during endoscopic submucosal dissection for gastric neoplasm. Surg. Endosc..

[47] Mentes O., Harlak A., Yigit T., Balkan A., Balkan M., Cosar A., Savaser A., Kozak O., Tufan T. (2008). Effect of intraoperative magnesium sulphate infusion on pain relief after laparoscopic cholecystectomy. Acta Anaesthesiol. Scand..

[48] Walia C., Gupta R., Kaur M., Mahajan L., Kaur G., Kaur B. (2018). Propofol sparing effect of dexmedetomidine and magnesium sulfate during BIS targeted anesthesia: A prospective, randomized, placebo controlled trial. J. Anaesthesiol. Clin. Pharmacol..

[49] Saadawy I.M., Kaki A.M., El Latif A.A.A., Abd-Elmaksoud A.M., Tolba O.M. (2010). Lidocaine vs. magnesium: Effect on analgesia after a laparoscopic cholecystectomy. Acta Anaesthesiol. Scand..

[50] Ko S.-H., Lim H.-R., Kim D.-C., Han Y.-J., Choe H., Song H.-S. (2001). Magnesium Sulfate Does Not Reduce Postoperative Analgesic Requirements. Anesthesiology.

[51] Hwang J.-Y., Na H.-S., Jeon Y.-T., Ro Y.-J., Kim C.-S., Do S.-H.I.V. (2010). infusion of magnesium sulphate during spinal anaesthesia improves postoperative analgesia. Br. J. Anaesth..

[52] Ryu J.-H., Kang M.-H., Park K.-S., Do S.-H. (2008). Effects of magnesium sulphate on intraoperative anaesthetic requirements and postoperative analgesia in gynaecology patients receiving total intravenous anaesthesia. Br. J. Anaesth..

[53] Ryu J.H., Koo B.W., Kim B.G., Oh A.Y., Kim H.H., Park D.J., Lee C.M., Kim S.T., Do S.H. (2016). Prospective, randomized and controlled trial on magnesium sulfate administration during laparoscopic gastrectomy: Effects on surgical space conditions and recovery profiles. Surg. Endosc..

[54] Bhatia A., Kashyap L., Pawar D.K., Trikha A. (2004). Effect of intraoperative magnesium infusion on perioperative analgesia in open cholecystectomy. J. Clin. Anesth..

[55] Shin H.-J., Kim E.-Y., Na H.-S., Kim T.K., Kim M.-H., Do S.-H. (2016). Magnesium sulphate attenuates acute postoperative pain and increased pain intensity after surgical injury in staged bilateral total knee arthroplasty: A randomized, double-blinded, placebo-controlled trial. Br. J. Anaesth..

[56] Sohn H.-M., Jheon S.-H., Nam S., Do S.-H. (2017). Magnesium sulphate improves pulmonary function after video-assisted thoracoscopic surgery. Eur. J. Anaesthesiol..

[57] Jaoua H., Zghidi S.M., Wissem L., Laassili S., Ammar N., Ali J., Darmoul S., Askri A., Khelifi S., Ben Maamer A. (2010). Effectiveness of intravenous magnesium on postoperative pain after abdominal surgery versus placebo: Double blind randomized controlled trial. Tunis. Med..

[58] Kumar M., Dayal N., Rautela R.S., Sethi A.K. (2013). Effect of intravenous magnesium sulphate on postoperative pain following spinal anesthesia. A randomized double blind controlled study. Middle East J. Anaesthesiol..

[59] Asadollah S., Vahdat M., Yazdkhasti P., Nikravan N. (2015). The effect of magnesium sulphate on postoperative analgesia requirements in gynecological surgeries. J. Turk. Soc. Obstet. Gynecol..

[60] Khafagy H.F., Ebied R.S., Osman E.S., Ali M.Z., Samhan Y.M. (2012). Perioperative effects of various anesthetic adjuvants with TIVA guided by bispectral index. Korean J. Anesthesiol..

[61] Ayoglu H., Karadeniz U., Kunduracilar Z., Ayoglu F.N., Erdemli O. (2005). The analgesic effect of magnesium sulfate and ketamine in patients undergoing laparoscopic cholecystectomy. Pain Clin..

[62] Koinig H., Wallner T., Marhofer P., Andel H., Hörauf K., Mayer N. (1998). Magnesium Sulfate Reduces Intra- and Postoperative Analgesic Requirements. Anesth. Analg..

[63] Çizmeci P., Ozkose Z. (2007). Magnesium Sulphate as an Adjuvant to Total Intravenous Anesthesia in Septorhinoplasty: A Randomized Controlled Study. Aesthetic Plast. Surg..

[64] Benhaj A.M., Barakette M., Dhahri S., Ouezini R., Lamine K., Jebali A., Ferjani M. (2008). Effect of intra and postoperative magnesium sulphate infusion on postoperative pain. Tunis Med..

[65] Seyhan T., Tuğrul M., Sungur M., Kayacan S., Telci L., Pembeci K., Akpir K. (2005). Effects of three different dose regimens of magnesium on propofol requirements, haemodynamic variables and postoperative pain relief in gynaecological surgery. Br. J. Anaesth..

[66] Frassanito L., Messina A., Vergari A., Colombo D., Chierichini A., Della Corte F., Navalesi P., Antonelli M. (2015). Intravenous infusion of magnesium sulfate and postoperative analgesia in total knee arthroplasty. Minerva Anestesiol..

[67] Olgun B., Oğuz G., Kaya M., Şavlı S., Eskiçırak H.E., Güney I., Kadıoğulları N. (2012). The effects of magnesium sulphate on desflurane requirement, early recovery and postoperative analgesia in laparascopic cholecystectomy. Magnes. Res..

[68] Kizilcik N., Köner Ö. (2018). Magnesium Sulfate Reduced Opioid Consumption in Obese Patients Undergoing Sleeve Gastrectomy: A Prospective, Randomized Clinical Trial. Obes. Surg..

[69] Oguzhan N., Gunday I., Turan A. (2008). Effect of magnesium sulfate infusion on sevoflurane consumption, hemodynamics, and perioperative opioid consumption in lumbar disc surgery. J. Opioid Manag..

[70] Zarauza R., Sáez-Fernández A.N., Iribarren M.J., Carrascosa F., Adame M., Fidalgo I., Monedero P. (2000). A Comparative Study with Oral Nifedipine, Intravenous Nimodipine, and Magnesium Sulfate in Postoperative Analgesia. Anesth. Analg..

[71] El Shal S.M., Lotfy E. (2017). Evaluation of effect of intravenous Magnesium Sulfate infusion on tourniquet induced hypertension and pain in arthroscopic knee surgery patients under epidural anesthesia. Egypt. J. Anaesth..

[72] Vicković S., Pjević M., Uvelin A., Pap D., Nikolić D., Lalić I. (2016). Magnesium Sulfate as an Adjuvant to Anesthesia in Patients with Arterial Hypertension. Acta Clin. Croat..

[73] Song J.W., Lee Y.-W., Yoon K.B., Park S.J., Shim Y.H. (2011). Magnesium Sulfate Prevents Remifentanil-Induced Postoperative Hyperalgesia in Patients Undergoing Thyroidectomy. Anesth. Analg..

[74] ElSersy H.E., Metyas M.C., Elfeky H.A., Hassan A.A. (2017). Intraoperative magnesium sulphate decreases agitation and pain in patients undergoing functional endoscopic surgery. Eur. J. Anaesthesiol..

[75] Mavrommati P.D., Gabopoulou Z.T., Papadimos C.N., Petsikopoulos M.G., Vrettou V.A., Konstantinidou M.G., Velmachou K.G. (2004). The perioperative infusion of low doses of magnesium sulfate reduces analgesic requirements in patients undergoing abdominal hernioplasty. Acute Pain.

[76] Kaya S., Kararmaz A., Gedik R., Turhanoğlu S. (2009). Magnesium sulfate reduces postoperative morphine requirement after remifentanil-based anesthesia. Med. Sci. Monit..

[77] Wilder-Smith O.H.G., Arendt-Nielsen L., Gaumann D., Tassonyi E., Rifat K.R. (1998). Sensory Changes and Pain After Abdominal Hysterectomy. Anesth. Analg..

[78] Tsaoui G., Nikopoulou A., Pezikoglou I., Birba V., Grosomanidis V. (2020). Implementation of magnesium sulphate as an adjunct to multinodal analgesic approach for perioperative pain control in lumbar laminectomy surgery: A randomised placebo-controlled clinical trial. Clin. Neurol. Neurosurg..

[79] Sousa A.M., Rosado G.M., Neto J.D.S., Guimarães G.M., Ashmawi H.A. (2016). Magnesium sulfate improves postoperative analgesia in laparoscopic gynecologic surgeries: A double-blind randomized controlled trial. J. Clin. Anesth..

[80] Shah P.N., Dhengle Y. (2016). Magnesium sulfate for postoperative analgesia after surgery under spinal anesthesia. Acta Anaesthesiol. Taiwanica.

[81] Tramèr M.R., Glynn C.J. (2007). An Evaluation of a Single Dose of Magnesium to Supplement Analgesia After Ambulatory Surgery: Randomized Controlled Trial. Anesth. Analg..

[82] Verki M.M., Porozan S., Motamed H., Fahimi M.A., Aryan A. (2019). Comparison the analgesic effect of magnesium sulphate and Ketorolac in the treatment of renal colic patients: Double-blind clinical trial study. Am. J. Emerg. Med..

[83] Sadrabad A.Z., Abarghouei S.A., Rad R.F., Salimi Y. (2020). Intravenous magnesium sulfate vs. morphine sulphate in relieving renal colic: A randomised clinical trial. Am. J. Emerg..

[84] El Sayed Z.M., Abouzeid A.E., El Sood A.I.A. (2019). Evaluating Effectiveness of Intravenous Magnesium Sulfate as a Treatment in Acute Renal Colic Patients Attending Suez Canal University Hospital Emergency Department. Med. J. Cairo Univ..

[85] Jokar A., Cyrus A., Babaei M., Taheri M., Almasi-Hashiani A., Behzadinia E., Yazdanbakhsh A. (2017). The Effect of Magnesium Sulfate on Renal Colic Pain Relief; a Randomized Clinical Trial. Emerg. Tehran. Iran.

[86] Majidi A., Derakhshani F. (2019). Intravenous Magnesium Sulfate for Pain Management in Patients with Acute Renal Colic; a Randomized Clinical Trial. Arch. Acad. Emerg. Med..

[87] Baratloo A., Mirbaha S., Kasmaei H.D., Payandemehr P., Elmaraezy A., Negida A. (2017). Intravenous caffeine citrate vs. magnesium sulfate for reducing pain in patients with acute migraine headache; a prospective quasi-experimental study. Korean J. Pain.

[88] Kandil M., Jaber S., Desai D., Cruz S.N., Lomotan N., Ahmad U., Cirone M., Burkins J., McDowell M. (2021). MAGraine: Magnesium compared to conventional therapy for treatment of migraines. Am. J. Emerg. Med..

[89] Frank L.R., Olson C.M., Shuler K.B., Gharib S.F. (2004). Intravenous magnesium for acute benign headache in the emergency department: A randomized double-blind placebo-controlled trial. Can. J. Emerg. Med..

[90] Ginder S., Oatman B., Pollack M. (2000). A prospective study of i.v. magnesium and i.v. prochlorperazine in the treatment of headaches. J. Emerg. Med..

[91] Cete Y., Dora B., Ertan C., Ozdemir C., Oktay C. (2005). A Randomized Prospective Placebo-Controlled Study of Intravenous Magnesium Sulphate vs. Metoclopramide in the Management of Acute Migraine Attacks in the Emergency Department. Cephalalgia.

[92] Shahrami A., Assarzadegan F., Hatamabadi H.R., Asgarzadeh M., Sarehbandi B., Asgarzadeh S. (2015). Comparison of Therapeutic Effects of Magnesium Sulfate vs. Dexamethasone/Metoclopramide on Alleviating Acute Migraine Headache. J. Emerg. Med..

[93] Demirkaya S., Vural O., Dora B., Topcuoglu M.A. (2001). Efficacy of intravenous magnesium sulfate in the treatment of acute migraine attacks. Headache J. Head Face Pain.

[94] Corbo J., Esses D., Bijur P.E., Iannaccone R., Gallagher E. (2001). Randomized clinical trial of intravenous magnesium sulfate as an adjunctive medication for emergency department treatment of migraine headache. Ann. Emerg. Med..

[95] Bigal E.M., Bordini C.A., Tepper S.J., Speciali J.G. (2002). Intravenous Magnesium Sulphate in the Acute Treatment of Migraine Without Aura and Migraine with Aura. A Randomized, Double-Blind, Placebo-Controlled Study. Cephalalgia.

[96] Brill S., Sedgwick P.M., Hamann W., Di Vadi P.P. (2002). Efficacy of intravenous magnesium in neuropathic pain. Br. J. Anaesth..

[97] Yousef A.A., Al-Deeb A.E. (2013). A double-blinded randomised controlled study of the value of sequential intravenous and oral magnesium therapy in patients with chronic low back pain with a neuropathic component. Anaesthesia.

[98] Karimi N., Razian A., Heidari M. (2021). The efficacy of magnesium oxide and sodium valproate in prevention of migraine headache: A randomized, controlled, double-blind, crossover study. Acta Neurol. Belg..

[99] Köseoglu E., Talaslioglu A., Gönül A.S., Kula M. (2008). The effects of magnesium prophylaxis in migraine without aura. Magnes. Res..

[100] Facchinetti F., Sances G., Borella P., Genazzani A.R., Nappi G. (1991). Magnesium Prophylaxis of Menstrual Migraine: Effects on Intracellular Magnesium. Headache J. Head Face Pain.

[101] Wang F., Eeden S.K.V.D., Ackerson L.M., Salk S.E., Reince R.H., Elin R.J. (2003). Oral Magnesium Oxide Prophylaxis of Frequent Migrainous Headache in Children: A Randomized, Double-Blind, Placebo-Controlled Trial. Headache J. Head Face Pain.

[102] Grazzi L., Andrasik F., Usai S., Bussone G. (2007). Magnesium as a preventive treatment for paediatric episodic tension-type headache: Results at 1-year follow-up. Neurol. Sci..

[103] Esfanjani A.T., Mahdavi R., Mameghani M.E., Talebi M., Nikniaz Z., Safaiyan A. (2012). The Effects of Magnesium, l-Carnitine, and Concurrent Magnesium–l-Carnitine Supplementation in Migraine Prophylaxis. Biol. Trace Elem. Res..

[104] Peikert A., Wilimzig C., Köhne-Volland R. (1996). Prophylaxis of Migraine with Oral Magnesium: Results from A Prospective, Multi-Center, Placebo-Controlled and Double-Blind Randomized Study. Cephalalgia.

[105] Pfaffenrath V., Wessely P., Meyer C., Isler H.R., Evers S., Grotemeyer K.H., Taneri Z., Soyka D., Bel G.H., Fischer M. (1996). Magnesium in the Prophylaxis of Migraine—A Double-Blind, Placebo-Controlled Study. Cephalalgia.

[106] Maizels M., Blumenfeld A., Burchette R. (2004). A Combination of Riboflavin, Magnesium, and Feverfew for Migraine Prophylaxis: A Randomized Trial. Headache J. Head Face Pain.

[107] Van Der Plas A.A., Schilder J.C., Marinus J., Van Hilten J.J. (2013). An Explanatory Study Evaluating the Muscle Relaxant Effects of Intramuscular Magnesium Sulphate for Dystonia in Complex Regional Pain Syndrome. J. Pain.

[108] Pickering G., Morel V., Simen E., Cardot J.-M., Moustafa F., Delage N., Picard P., Eschalier S., Boulliau S., DuBray C. (2011). Oral magnesium treatment in patients with neuropathic pain: A randomized clinical trial. Magnes. Res..

[109] Russell I.J., Michalek E.J., Flechas J.D., Abraham E.G. (1995). Treatment of fibromyalgia syndrome with Super Malic: A randomized, double blind, placebo controlled, crossover pilot study. J. Rheumatol..

[110] Bagis S., Karabiber M., As I., Tamer L., Erdogan C., Atalay A. (2012). Is magnesium citrate treatment effective on pain, clinical parameters and functional status in patients with fibromyalgia?. Rheumatol. Int..

[111] Coudray C., Rambeau M., Feillet-Coudray C., Gueux E., Tressol J.C., Mazur A., Rayssiguier Y. (2005). Study of magnesium bioavailability from ten organic and inorganic Mg salts in Mg-depleted rats using a stable isotope approach. Magnes. Res..

[112] Uysal N., Kizildag S., Yuce Z., Guvendi G., Kandis S., Koc B., Karakilic A., Camsari U.M., Ates M. (2019). Timeline (Bioavailability) of Magnesium Compounds in Hours: Which Magnesium Compound Works Best?. Biol. Trace Elem. Res..

[113] Lindberg J.S., Zobitz M.M., Poindexter J.R., Pak C.Y. (1990). Magnesium bioavailability from magnesium citrate and magnesium oxide. J. Am. Coll. Nutr..

[114] Walker A.F., Marakis G., Christie S., Byng M. (2003). Mg citrate found more bioavailable than other Mg preparations in a randomised, double-blind study. Magnes. Res..

[115] Brilli E., Khadge S., Fabiano A., Zambito Y., Williams T., Tarantino G. (2018). Magnesium bioavailability after administration of sucrosomial^®^ magnesium: Results of an ex-vivo study and a comparative, double-blinded, cross-over study in healthy subjects. Eur. Rev. Med Pharmacol. Sci..

[116] Blancquaert L., Vervaet C., Derave W. (2019). Predicting and Testing Bioavailability of Magnesium Supplements. Nutrients.

[117] Duale C., Cardot J.-M., Joanny F., Trzeciakiewicz A., Martin E., Pickering G., DuBray C. (2018). An Advanced Formulation of a Magnesium Dietary Supplement Adapted for a Long-Term Use Supplementation Improves Magnesium Bioavailability: In Vitro and Clinical Comparative Studies. Biol. Trace Elem. Res..

[118] Ranade V.V., Somberg J.C. (2001). Bioavailability and Pharmacokinetics of Magnesium after Administration of Magnesium Salts to Humans. Am. J. Ther..

[119] Treede R.-D., Rief W., Barke A., Aziz Q., Bennett M.I., Benoliel R., Cohen M., Evers S., Finnerup N.B., First M.B. (2015). A classification of chronic pain for ICD-11. Pain.

[120] Benyamin R., Trescot A., Datta S., Buenaventura R., Adlaka R., Sehgal N., Glaser S.E., Vallejo R. (2008). Opioid complications and side effects. Pain Physician.

[121] Lee M., Silverman S.M., Hansen H., Patel V.B., Manchikanti L. (2011). A comprehensive review of opioid-induced hyperalgesia. Pain Physician.

[122] Office of Dietary Supplements—Magnesium, n.d. https://ods.od.nih.gov/factsheets/Magnesium-HealthProfessional/.

[123] Nieves J.W. (2005). Osteoporosis: The role of micronutrients. Am. J. Clin. Nutr..

[124] Rachner T.D., Khosla S., Hofbauer L.C. (2011). Osteoporosis: Now and the future. Lancet.

[125] Castiglioni S., Cazzaniga A., Albisetti W., Maier J.A.M. (2013). Magnesium and Osteoporosis: Current State of Knowledge and Future Research Directions. Nutrients.

[126] Alfrey A.C., Miller N.L. (1973). Bone Magnesium Pools in Uremia. J. Clin. Investig..

[127] Huang E.P. (1998). Synaptic plasticity: Going through phases with LTP. Curr. Biol..

[128] Inagaki T., Begum T., Reza F., Horibe S., Inaba M., Yoshimura Y., Komatsu Y. (2008). Brain-derived neurotrophic factor-mediated retrograde signaling required for the induction of long-term potentiation at inhibitory synapses of visual cortical pyramidal neurons. Neurosci. Res..

[129] Morel V., Joly D., Villatte C., DuBray C., Durando X., Daulhac L., Coudert C., Roux D., Pereira B., Pickering G. (2016). Memantine before Mastectomy Prevents Post-Surgery Pain: A Randomized, Blinded Clinical Trial in Surgical Patients. PLoS ONE.

[130] Morel V., Joly D., Villatte C., Pereira B., Pickering G. (2018). Preventive effect of oral magnesium in postmastectomy pain: Protocol for a randomised, double-blind, controlled clinical trial. BMJ Open.

[131] Hanus M., Lafon J., Mathieu M. (2003). Double-blind, randomised, placebo-controlled study to evaluate the efficacy and safety of a fixed combination containing two plant extracts (Crataegus oxyacantha and Eschscholtzia californica) and magnesium in mild-to-moderate anxiety disorders. Curr. Med Res. Opin..

[132] Wienecke E., Nolden C. (2016). Langzeit-HRV-Analyse zeigt Stressreduktion durch Magnesiumzufuhr. MMW Fortschr. Med..

